# Increasing the melting temperature of VHH with the in silico free energy score

**DOI:** 10.1038/s41598-023-32022-8

**Published:** 2023-03-25

**Authors:** Yusuke Tomimoto, Rika Yamazaki, Hiroki Shirai

**Affiliations:** 1 Applied Research and Operations, Astellas Pharma Inc., Tsukuba city, Ibaraki 305-8585 Japan; 2 Present Address: Riken Center for Computational Science, Nihonbashi 1-Chome Mitsui Building, 15th floor, 1-4-1 Nihonbashi, Tsukuba, 103-0027 Japan

**Keywords:** Biochemistry, Computational biology and bioinformatics, Protein analysis, Protein design

## Abstract

VHH, the antigen-binding fragment of a heavy chain-only antibody, is a useful component of antibody-based therapeutics. Thermal stability, represented by the melting temperature (Tm), is one of the key factors affecting the developability of antibody-based therapeutics. In this study, we examined whether the in silico free energy score dStability can be used to design mutants with improved Tm compared to the anti-lysozyme VHH, D3-L11. After verifying that exhaustive mutagenesis was inefficient for improving Tm, we performed a two-round rational approach that combined dStability calculations with a small number of experiments. This method improved the Tm by more than 5 °C in several single mutants including A79I. It reduced the affinity for the antigen by less than 1.6-fold. We speculate that stabilization of A79I required exquisite compatibility among neighboring residues to fill in the internal cavity in the protein. Given that we identified only one mutation that could simultaneously improve Tm and almost maintain affinity, we concluded that achieving both is extremely difficult, even with single mutations that are not located in the paratope. Therefore, we recommend using a variety of approaches when trying to achieve such a feat. Our method will be a useful complementary approach to other existing methods.

## Introduction

Antibody-based therapeutics have emerged as best-selling medicines in recent years. VHH is the antigen-binding fragment of a heavy chain-only antibody produced by camelids^1^ and has become a useful component of antibody-based therapeutics^2^. For example, the anti-von Willebrand factor bivalent VHH Caplacizumab is approved as a therapeutic drug for acquired thrombotic thrombocytopenia purpura^3^. Further, the anti-TNFα/anti-HSA bispecific VHH Ozoralizumab is approved for the treatment of rheumatoid arthritis^4^. To be effective, antibody-based therapeutics need to strike a balance among developability, immunogenicity and affinity.

Thermal stability represented by the melting temperature (Tm) is one of the key factors affecting their developability. Although VHH is considered more stable than conventional antibody variable domain composed of heavy and light chains, the thermal stability of VHH depends on its clone^5,6^. Various approaches to increase the Tm of VHH have been proposed, including disulfide bond introduction^7–12^, complementarity-determining region (CDR) grafting^13,14^, random mutagenesis^15–17^, mutation to consensus sequence^18–20^, and backmutation to animal sequence after humanization^21^. Further, methods such as circular dichroism^22^, differential scanning calorimetry^23,24^ and differential scanning fluorometry (DSF)^25,26^ have been used to evaluate the Tm. Although introducing a disulfide bond is a powerful approach for raising the Tm^7–12^, it usually leads to disulfide shuffling, which prevents refolding at the purification step^11^, and contamination with unwanted, erroneously shuffled products makes the protein formulation less developable.

Antigen recognition in so-called conventional antibodies produced in species such as humans and mice occurs through a total of 6 CDRs, of which 3 CDRs are derived from the heavy chain and the other 3 from the light chain. The non-CDR antibody variable region is called the framework region (FR). CDR grafting is usually used for humanization: grafting CDRs from animal antibodies onto the FRs of human antibodies to reduce the antibodies’ immunogenicity. VHH has a structure similar to the heavy chain of the variable region of a conventional antibody, and mainly uses three CDRs to bind antigens. CDR grafting of VHH is a protein engineering method used to graft CDR onto another VHH’s FR. This technique is useful for not only humanization but also stabilization of VHH. CDR grafting onto a more stable VHH’s FR is performed for the latter purpose. However, incompatibility between the CDR and FR sometimes decreases Tm. In addition, CDR grafting usually decreases the VHH’s affinity for an antigen^14^. Therefore, whether CDR grafting improves the Tm and retains antigen binding affinity depends on the clone sequence.

The mutagenesis approach can be divided into two types: random mutagenesis^15–17^ and site-directed mutagenesis^18–21^. The advantage of the former is that it enables the introduction of mutations that are theoretically unpredictable. However, because random mutagenesis does not always lead to the generation of all possible mutations, it may miss more effective mutations. Another potential advantage of random mutagenesis is that it makes it possible to easily introduce multiple mutations at once. However, as artificial mutations increase the risk of reducing affinity and enhancing immunogenicity, it would be more desirable to improve Tm by introducing a small number of mutations, ideally a single mutation.

Two methods have been proposed for site-directed mutagenesis. The first is back mutation, a method used to return the amino acid in FR that interacts with CDR to that in the original antibody. As mentioned above, CDR grafting often reduces affinity, making back mutation necessary. Vincke et al. reported that back mutation improved Tm^21^. However, this approach is limited to those who want to recover the Tm that was reduced by CDR grafting. The second method of site-directed mutagenesis is to mutate a rare amino acid into a consensus amino acid at the same position^18–20^. This approach takes advantage of the fact that each residue of VHH has a different propensity for amino acids. However, mutations that produce consensus amino acids are not optimal for all VHH, and more effective mutations may be missed. Thus, there is currently no optimal method, indicating the need for an alternative or complementary approach.

One complimentary approach could be to employ the change in Gibbs free energy (ΔG). As ΔG between the folded and unfolded states of a protein determines the thermal stability of the native fold, a change in ΔG (ΔΔG) due to mutation should reflect the thermal stability of the mutant. Thus, ΔΔG is often used to predict protein stability^27^. We hypothesized that ΔΔG could be a useful indicator of a change in Tm (ΔTm). In this paper, we used MOE molecular modeling software^28^ to calculate the dStability score as ΔΔG, and examined whether we could enhance the Tm of the anti-lysozyme VHH, D3-L11^24,29^, using the dStability score. After verifying that exhaustive mutagenesis was inefficient for improving Tm, we performed a two-round rational approach that combined dStability calculations with a small number of experiments focusing on high throughput and low sample volume.

In a recent study, Akiba et al. performed comprehensive analysis of the interaction between D3-L11 and its antigen^24^. They generated eleven single mutants by replacing an amino acid residue located in the paratope of D3-L11 with alanine. Of these, four mutants (E32A, Y52A, R106A and S113A) showed increased Tm by less than 3 °C, but more than ten-fold lower affinity. Only one mutant (K101A) showed increased affinity for the antigen; however, its Tm was reduced by more than 3 °C. Five mutants (H54A, T55A, Y102A, P104A and F107A) showed decreased Tm and ten-fold lower affinity, and the remaining mutant (D115A) showed decreased Tm and less than ten-fold lower affinity. This indicates that it is extremely difficult to simultaneously improve the Tm and maintain the affinity of D3-L11 with a single mutation in the paratope. Using our method, only mutations located outside the paratope successfully improved Tm. We expected that these mutations would not decrease the affinity. Thus, we examined whether the group of mutants with improved Tm also maintained affinity by measuring the affinity of our first and second-round mutants for the antigen lysozyme using surface plasmon resonance (SPR) and enzyme-linked immunosorbent assay (ELISA). Structure-based analysis was performed using modeling and docking studies.

## Results

### Comprehensive site-directed mutagenesis

**Figure 1:**
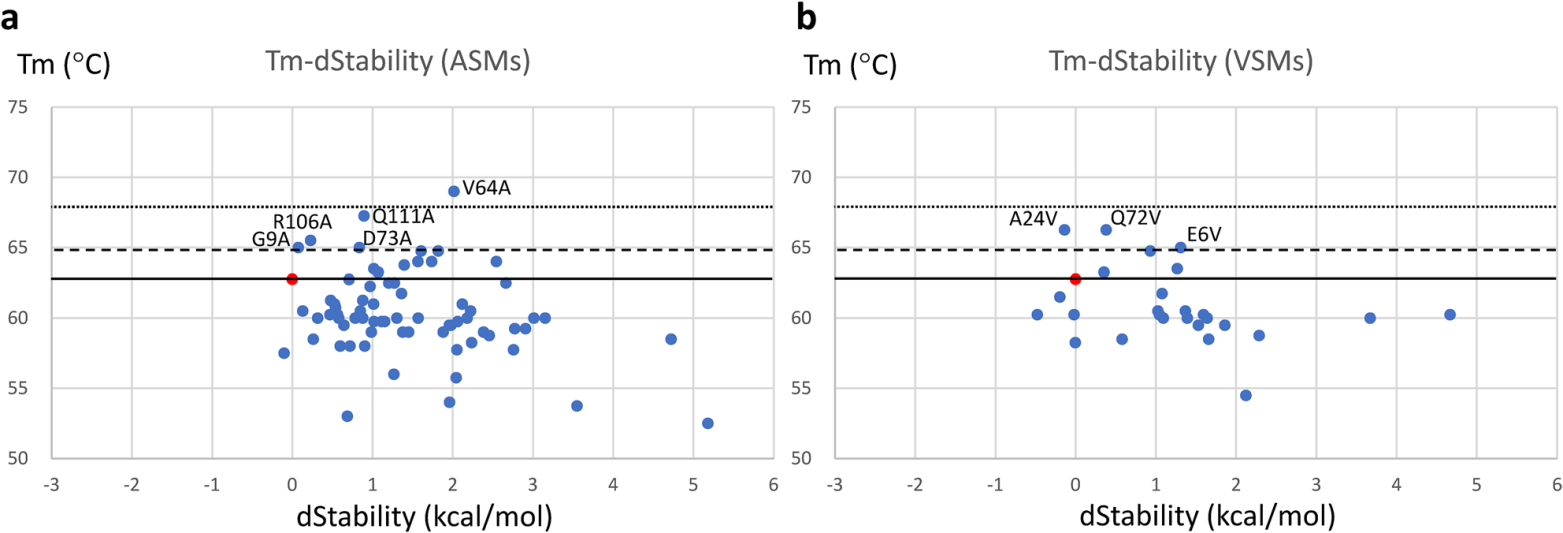
Scatter plots of melting temperature (Tm) measured by differential scanning fluorometry and dStability calculated using MOE software for alanine-substituted mutants (ASMs) (**a**) and valine-substituted mutants (VSMs) (**b**). Data points in red show wild type. The broken and dotted lines show increases in Tm by 2 °C and 5 °C, respectively.

We first used a comprehensive site-directed mutagenesis approach to understand the degree of difficulty of enhancing Tm. We made constructs of alanine-substituted mutants (ASMs) for all residues except for the alanine residue. We theorized that substituting to alanine might create voids inside the protein and make it unstable. However, substitution with an amino acid that is too large may also cause destabilization due to steric hindrance. Thus, we also performed comprehensive mutagenesis by substituting to valine, the second smallest hydrophobic amino acid. We made constructs of valine-substituted mutants (VSMs) for all interior residues except for the valine residue. We attempted to construct a total of 115 ASMs by replacing all except for alanine residues and a total of 35 VSMs by replacing all except for protein surface and valine residues. However, the DNA sequences of 39 ASMs and 9 VSMs could not be confirmed using a sequencer, probably due to failure of inverse PCR. We expressed and purified for the remaining 76 ASMs and 26 VSMs. Among these, the purified protein concentrations of 26 ASMs and 9 VSMs were below 0.35 mg/mL, probably due to their low expression levels. We measured the Tm of these poorly expressed mutants using DSF without dilution. The remaining highly expressed 50 ASMs and 17 VSMs were diluted to 0.35 mg/mL before measurement of Tm using DSF. All of the poorly expressed mutants and most of the highly expressed mutants showed decreased Tm. Only 5 mutants (G9A, V64A, D73A, R106A and Q111A) of 76 ASMs and 3 mutants (E6V, A24V and Q72V) of 26 VSMs showed an increase in Tm by more than 2 °C (Fig. 1). Only one ASM and no VSMs showed an increase in Tm by more than 5 °C. Pearson’s correlation coefficients between the Tm and dStability score of ASMs and VSMs were as low as − 0.30 and − 0.32, respectively. From these results we concluded that comprehensive site-directed mutagenesis was not effective for improving Tm, and that the dStability score was not useful to quantitatively predict the Tm of ASMs and VSMs. However, we noted that a large positive dStability score led to destabilization such that ASMs with a dStability score of 2.6 or higher and VSMs with a dStability score of 1.5 or higher always showed decreased Tm. In addition, we noted that two residues with extremely positive dStability, C22 and C96, formed a disulfide bond, which contributes significantly to the stabilization of VHH. These observations led us to hypothesize that mutations with extremely negative dStability values could be suitable positions at which to introduce mutations. Therefore, we performed a two-round rational approach that combined dStability calculations with a small number of experiments.

### Two-round rational approach

**Figure 2:**
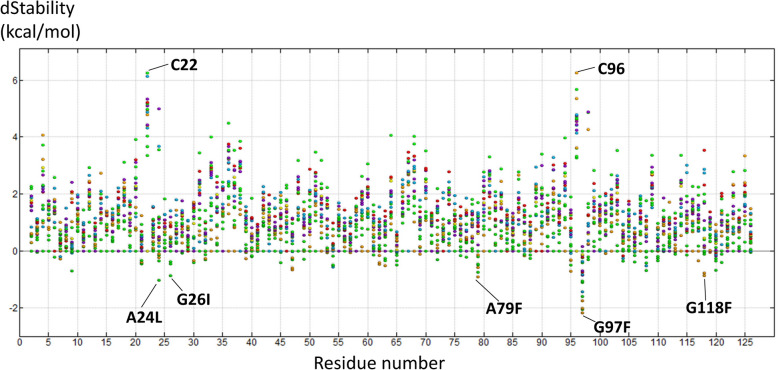
dStability scores of all 2375 single mutants and wild type. The x-axis shows the residue number. The dStability score of pristine amino acids at each position is zero. A smaller dStability score is predictive of a more stable mutation. The color of each data point is based on the characteristic of the amino acids: green points show aliphatic (G, A, V, L, I, M, P), yellow cysteine (C), orange aromatic (F, W, Y), red acidic (D, E), blue basic (R, K, H), and purple all other hydrophilic (S, T, N, Q) amino acids. The top five mutations selected at first-round and C22–C96, which form a disulfide bond, are labeled in the figure.

**Figure 3:**
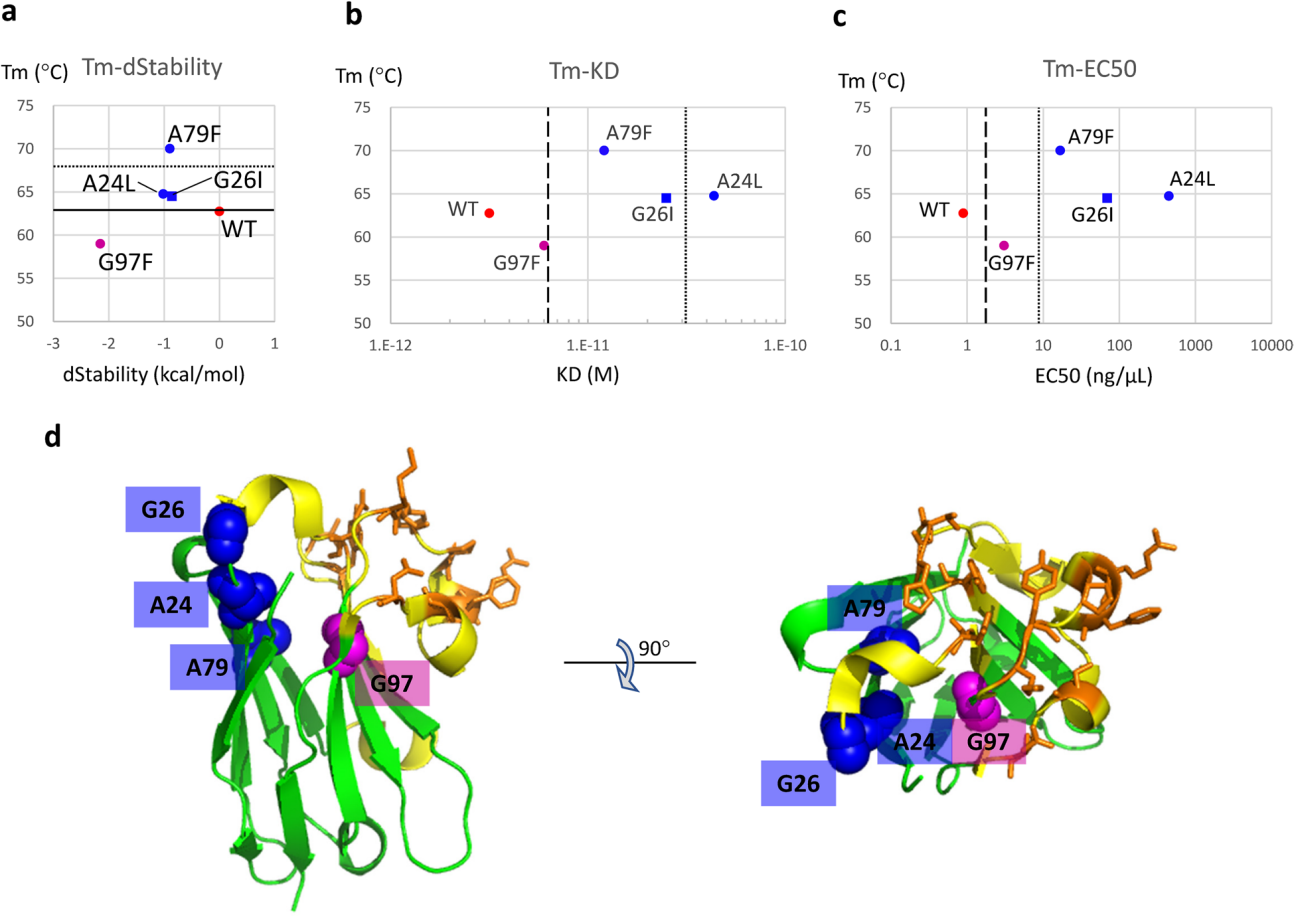
Results from the first-round of mutations. (**a**) Scatter plot of the melting temperature (Tm) obtained by DSF versus dStability calculated by MOE. The solid line shows the Tm observed in wild type and dotted line shows an increase in Tm by 5 °C. The three mutants (A24L, G26I, A79F) in the top-left corner of the graph are more stable while the remaining mutant (G97F) is less stable than wild type. The red circle shows wild type (WT), blue circles (A79F and A24L) indicate mutants in which Tm increased through the proposed “filling the void” mechanism, and the blue square (G26I) indicates a mutant in which Tm increased through the proposed “fixed flexible loop” mechanism (see Discussion). The magenta circle (G97F) indicates the mutant with decreased Tm. (**b**) Scatter plot of melting temperature (Tm) obtained by DSF versus K_D_ values obtained by SPR. The colors and symbols represent the same mutants as those in (**a**). The broken and dotted lines show K_D_ values two-fold and ten-fold lower than that in wild type, respectively. (**c**) Scatter plot of melting temperature (Tm) obtained by DSF versus EC50 values obtained by ELISA. The colors and shapes represent the same mutants as those in (**a**). The broken and dotted lines show K_D_ values two-fold and ten-fold lower than that in wild type, respectively. (**d**) Overall view of the residues selected in the first-round of mutations. These residues are shown in a space-filling model. Residues at which mutation increased Tm (A24, G26 and A79) are colored blue and the residue at which mutation decreased Tm (G97) is colored magenta. Residues in the paratope are shown as orange sticks. Yellow and green ribbons show the CDR and framework, respectively.

**Figure 4:**
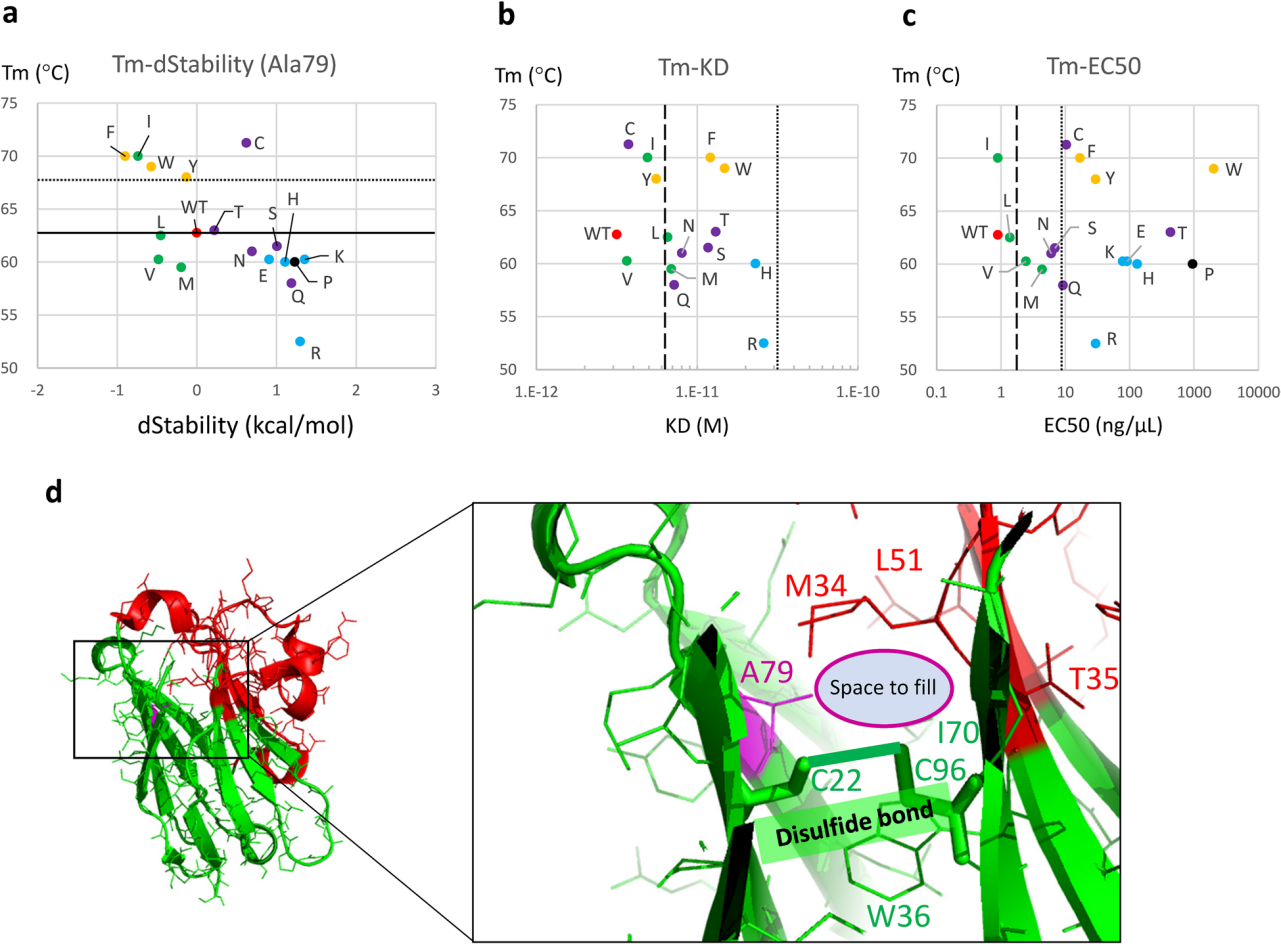
Results from the second round of A79-series mutations. (**a**) Scatter plot of the melting temperature (Tm) versus dStability scores of A79 mutants. The solid line shows the Tm observed in wild type and dotted line shows an increase in Tm by 5 °C. The color of each data point is based on the characteristic of the amino acids: red shows wild type (WT, alanine), yellow shows aromatic amino acids (F, W, Y), green shows aliphatic amino acids (I, L, V, M), black shows proline (P), blue shows charged amino acids (E, H, K, R), and purple shows all other uncharged polar (S, T, N, Q, C) amino acids. Five (F, I, W, Y, C) of the 17 purified mutants were stable. (**b**) Scatter plot of melting temperature (Tm) obtained by DSF versus K_D_ values obtained by SPR. The color scheme is the same as that in (**a**). The broken and dotted lines show K_D_ values two-fold and ten-fold lower than that in wild type, respectively. (**c**) Scatter plot of melting temperature (Tm) obtained by DSF versus EC50 values obtained by ELISA. The color scheme is the same as that in (**a**). The broken and dotted lines show K_D_ values two-fold and ten-fold lower than that in wild type, respectively. (**d**) Overall (left) and close-up (right) view of A79 and its surrounding residues. A79 is shown as thin magenta-colored lines. Cysteines that form a disulfide bond are shown as green sticks. Other residues are depicted as thin lines.

To select mutations for experimental validation, we calculated the dStability score of all simulated single mutations (Fig. 2). Among a total of 125 simulated mutations with the lowest dStability scores at each residue, we selected the five mutations (A24L, G26I, A79F, G97F and G118F) with the lowest dStability scores as our first-round of mutations. We then generated these five mutations experimentally and measured their Tm. While we failed to make an expression plasmid of G118F using inverse PCR, capillary electrophoresis confirmed that we had successfully expressed and purified the remaining 4 mutants (Supplementary Figure S1). Of these, 3 mutants (A24L, G26I and A79F) showed enhanced Tm, while the remaining mutant, G97F, showed decreased Tm (Fig. 3a). As the A79F mutant had the most improved Tm (increased by more than 5 °C), we next mutated the A79 residue into all other amino acids to obtain our second-round of mutations. Although we failed to construct plasmids of A79G and A79D using inverse PCR, capillary electrophoresis confirmed that we had successfully expressed and purified 14 mutants (Y, W, I, L, V, C, N, Q, E, H, R, E, K, P mutants at A79), as evidenced by the presence of a single band for each. High molecular weight impurities were identified for the A79S and A79T mutants; however, the position of the bands suggested that they were dimers of VHH (Supplementary Figure S1). Therefore, the Tm and affinity of all mutants except for A79G and A79D were measured. The results obtained for our second-round of A79-series mutations, together with A79F, are shown in Fig. 4a. Four mutations (A79I, A79W, A79Y and A79C), like A79F, increased the Tm by more than 5 °C. Pearson’s correlation coefficient between the Tm and dStability score of A79 mutants was as high as − 0.64. The Tm and dStability values of wild type and mutants generated in this study are summarized in Supplementary Table S1.

### Affinity for the antigen lysozyme

Based on previous evidence^24^, we predicted that it would be difficult to simultaneously increase the Tm of D3-L11 and maintain its affinity for its antigen after introducing a mutation into the paratope. We performed SPR to measure the K_D_ between the antigen lysozyme and VHHs for the wild type and four first-round mutants (A24L, G26I, A79F and G97F). The sensorgrams are shown in Supplementary Figure S2 and the K_D_ values are summarized in Supplementary Table S2. The scatter plot of Tm versus K_D_ is shown in Fig. 3b. We also performed ELISA to measure the EC50 value for these VHHs. The binding curves are shown in Supplementary Figure S5 and the EC50 values are summarized in Supplementary Table S2. The scatter plot of Tm versus EC50 is shown in Fig. 3c. Affinity was attenuated in all four mutants, but to different degrees in SPR and ELISA. In Fig. 3b,c a two-fold or ten-fold decrease in affinity is indicated by the dashed and dotted lines, respectively. The three mutants (A24L, G26I and A79F) with increased Tm showed a greater decrease in affinity than G97F in both SPR and ELISA.

We also performed SPR and ELISA for the second-round mutants. The raw data are shown in Supplementary Figure S3–S5 and Table S2, and the scatter plots are shown in Fig. 4b, c. We could not determine the K_D_ for A79E, A79K and A79P using SPR due to insufficient quantities of these mutants. The A79I mutant completely retained its EC50 value using ELISA and only a 1.6-fold reduction in affinity using SPR. In the remaining cases, the affinity was reduced to different degrees. According to the K_D_ values calculated using SPR, all A79 mutants showed a less than ten-fold decrease in affinity, with I, C, Y and V mutants showing a less than two-fold reduction. According to the EC50 values calculated using ELISA, I, L, V, M, N and S mutants showed a less than ten-fold decrease in affinity (Fig. 4c). From these results, the point mutations we found tended to improve Tm more than and reduce affinity less than mutations on the paratope. Our method successfully increased the Tm by more than 5 °C while decreasing affinity (increasing K_D_) by less than ten-fold as measured by SPR in five single mutants (A79I, A79C, A79F, A79Y and A79W). Of note, the A79I mutant showed an increase in Tm by more than 5 °C and unchanged EC50 using ELISA and only a 1.6-fold reduction in affinity using SPR. However, since we identified only one mutant (A79I) that simultaneously showed improved Tm and almost maintained affinity, we conclude that even for single mutations not located in the paratope, it is generally difficult to improve Tm and maintain affinity simultaneously.

### Modeling

To investigate why some mutants showed improved Tm, some showed little decrease in affinity, and why it is generally difficult to both improve Tm and maintain affinity, we conducted a tertiary structure analysis using modeling and docking studies. First, we built three-dimensional structural models of the first and second-round mutants using MOE^28^ and compared them with each other and with the wild type VHH structure. To offset the effects of computational modeling, we also constructed a model structure of wild-type VHH, and compared it with the crystal structure of wild-type VHH. Supplementary Figure S6 shows the main chain structural differences among the first and second-round mutants and the model and crystal structures of wild type VHH. We observed a large difference between the crystal structure of wild type and the model structures including that of wild type. In fact, a difference of 0.92 Å in Root Mean Square Deviation (RMSD) was noted between the crystal and model structure of the wild type VHH, compared to a maximum difference of 0.8 Å between the model structures (Supplementary Figure S7). The smaller difference between the model structures compared to that between the crystal structure and model structure of wild type VHH protein illustrates the difficulty of identifying the effects of mutating just one residue in the model structure. However, with this limitation in mind, comparison of only model structures allowed us to clearly classify them into two clusters. The first cluster, which we called class-1, included the wild type model structure, G26I, G97F, and I, C, L, V, H, E, K, R, T, N and P mutants at A79. The other cluster, which we called class-2, included A24L and F, Y, W, M, Q and S mutants at A79. Since wild type VHH belongs to class-1, we propose that class-2 mutants may undergo relatively greater structural changes upon mutation than class-1 mutants.

We then investigated how these structural differences relate to Tm and affinity. A total of 7 mutants (A24L, G26I, A79F, A79I, A79Y, A79W and A79C) showed an increase in Tm by more than 1.5 °C. Of the seven class-2 mutants, four (A24L, A79F, A79Y and A79W) had improved Tm (57.1%), while only three (G26I, A79I and A79C) of the 13 class-1 mutants had improved Tm (23.1%). This suggests that improved Tm may be accompanied by a structural change in mutation. In terms of affinity, a total of six mutants (G97F, A79I, A79C, A79L, A79V and A79Y) showed a less than two-fold decrease in affinity according to either K_D_ or EC50. Of the 13 mutants in class-1, five mutants (A79I, A79C, A79L, A79V and G97F) showed a less than two-fold decrease in affinity (38.5%), while only one (A79Y) of the 7 mutants in class-2 showed such a decrease in affinity (14.3%). This suggests that retaining affinity tends not to be accompanied by a structural change. Thus, based on our modeling study, the reason why it is difficult to both improve Tm and maintain affinity is the conflicting effect of structural changes, which are desirable for improving Tm, but undesirable for maintaining affinity.

Next, we examined whether point mutations alter local but distinct structural features, especially near the paratope. To do this, we used the sequence analysis tool, JOY^30^ (Supplementary Figure S8), which annotates protein sequence alignments with three-dimensional (3D) structural features. It was developed to display 3D structural information in a sequence alignment and to help understand the conservation of amino acids in their specific local environments. Distinct structural features that can be analyzed with JOY include solvent accessibility (accessible, inaccessible), secondary structure (alpha-helix, beta-sheet, 3–10 helix), main-chain conformation (positive phi, cis peptide, disulfide bond), and hydrogen bond (hydrogen bond to the other side chain, main chain amide, main chain carbonyl). As shown in Supplementary Figure S8, we found the local structural features to be similar for all structures. Although some differences were noted in the presence or absence of 3–10 helices, changes in solvent accessibility, and β-sheet length, the degree of these differences was comparable to that between the crystal and model structures of wild type VHH. No distinguishing features were observed between mutants whose model structure was classified as class-1 and 2. In addition, there were no common features among the seven mutants with improved Tm, nor among the six mutants that showed less than a two-fold decrease in affinity.

We then mapped the positions of the paratopes on this alignment to investigate whether point mutations altered the local structural features of the paratopes. Structural changes in the paratope were observed at two positions. One paratope residue, E32, is solvent accessible only for the wild type crystal structure and the A79P model structure. The second paratope residue, S113, constructs a 3–10 helix and is solvent inaccessible only in the wild type crystal structure. However, it is unlikely that these feature differences were caused by amino acid mutations, because even native-VHH crystal and model structures differ in these features.

Therefore, it was difficult to interpret our experimental data using the local structural features detected using JOY analysis. It is interesting that while small structural changes over the entire protein, as in the class-1 and class-2 classifications, were useful for interpreting the experimental results, changes in local but distinct structural features were not. However, based on these results alone, it is impossible to determine whether the point mutation was insufficient to change distinct features of the local structure or whether the lack of change is a consequence of poor modeling accuracy.

### Docking

Next, we performed docking simulations between lysozyme and VHHs using FRODOCK^31,32^. The structure with the best score in each docking simulation was selected, and the main chain conformations of VHH were superposed (Supplementary Figure S9). Docking poses of all mutants and wild type were similar to each other. In Supplementary Figure S9a, class-1 and class-2 structures are represented by red and cyan lines, respectively. We did not observe any notable differences between class-1 and class-2 VHHs. From the inputted VHH-antigen complex structures, MOE outputted a total of 33 scores indicating the degree to which the 11 paratope residues contribute to hydrophobic interactions, ionic interactions and hydrogen bonding (Supplementary Table S3). Compared to the difference between the crystal and model structures of the wild type VHH, no significant difference was observed between the mutants or between the mutant and wild type VHH. Supplementary Figure S10 shows an overlay of the structures of the A79I mutant (class-1 representative), A79W mutant (class-2 representative), and wild type crystal and model structures. Both A79I and A79W showed an increase in Tm by more than 5 °C; however, while A79I almost maintained affinity, A79W showed greatly reduced affinity. In the superposition of the four structures, the rotamers of the 11 paratope residues were consistent. The difference in the paratope residue between A79I and A79W was not remarkable compared to the difference between the wild type crystal structure and model structure.

Thus, the docking study was not useful for interpreting our experimental results. This may be because the differences in the model structures of the point mutants were small, and the differences in the docking modes using these were even smaller and could not be detected.

## Discussion

There has been a recent shift from using naked antibodies to engineered antibodies in antibody drugs, making developability an increasingly important factor in the establishment of these therapeutics. Thermal stability is a major factor affecting developability. Despite reports of various ways to enhance thermal stability, no studies have comprehensively investigated whether or not a simple alanine substitution can elevate Tm. In this study, we constructed comprehensive site-directed ASMs for all residues except the alanine residue. Theoretically, however, changing a residue located inside a protein to alanine, which is the second smallest amino acid, could create voids, thereby destabilizing the VHH. Therefore, we also constructed comprehensive site-directed VSMs for all interior residues except for valine. We were able to construct a total of 76 ASMs and 26 VSMs, and express and purify their proteins. Among these, only one ASM and no VSMs showed an increase in Tm by more than 5 °C. We concluded that comprehensive site-directed mutagenesis was not effective for improving Tm, and that the dStability score was not useful for quantitatively predicting the Tm of ASMs and VSMs. However, based on our observations, we hypothesized that mutations with extremely negative dStability values could be suitable positions at which to introduce mutations.

By investigating whether the Tm improved among the 5 mutations with the lowest dStability score that we selected in the first-round of mutations in the rational approach, we found that 3 mutations showed an increase in Tm by more than 1.5 °C (Fig. 3a). Better still, a mutation at the A79 residue increased the Tm by more than 5 °C. As the A79F mutant was the most stable and showed the greatest elevation in Tm, we then mutated the A79 residue into all other amino acids to obtain our second-round of mutations. The four mutations (A79I, A79W, A79Y, A79C) together with A79F showed an increase in Tm of more than 5 °C demonstrates that mutations with extremely low dStability scores could be useful for predicting increased Tm (Fig. 4a). The correlation coefficient between the Tm and dStability in mutants of the A79 residue was as high as − 0.64, and the absolute value was greater than that observed in comprehensive site-directed mutagenesis.

Akiba et al. used various analytical methods to analyze the physicochemical characteristics of D3-L11 and lysozyme binding at the residue level^24^. They constructed a total of 11 mutants with mutations in the paratope and measured their affinity for lysozyme and D3-L11 stability. Among these, four mutants, E32A, Y52A, R106A and S113A, showed an increase in Tm of less than 3 °C and more than ten-fold decrease in affinity for lysozyme. In contrast, while the K101A mutant showed enhanced affinity for lysozyme, its Tm was significantly reduced. These findings suggest that it may not be possible to both improve Tm and maintain affinity by mutating amino acid residues in the paratope to alanine. In the present study, none of the mutations introduced in our first-round of mutations were in the paratope. Therefore, we were motivated to investigate whether our method could enhance Tm without reducing affinity.

The steps of our two-round method are (1) Calculate the dStability score for all possible mutations, (2) Select a limited number of mutations with the lowest dStability scores among the simulated mutations with the lowest dStability scores at each residue as the first-round of mutations, (3) Prepare the mutants and measure their Tm experimentally, (4) Identify the mutant with the most improved Tm and substitute the residue with the remaining 18 amino acids to obtain the second-round of mutations, (5) Measure the K_D_ value using SPR and EC50 value using ELISA for the first and second-round mutants.

Using this method, we successfully generated A79I, which showed an increase in Tm by more than 5 °C and completely retaining EC50 using ELISA and only a 1.6-fold reduction in affinity according to the K_D_ value calculated using SPR. A79C, A79Y, A79W and A79F also showed an increase in Tm by more than 5 °C and a less than ten-fold reduction in affinity in terms of the K_D_ value.

Some studies have reported that changes to consensus amino acids improve the Tm^18–20^. Since we did not exhaustively investigate consensus mutations, we cannot conclude whether such a technique would be more or less effective than our method. However, if we had adopted the consensus method at the A79 residue, we would have created an A79V mutant, which we showed had a lower Tm, and would not have generated the successful mutant, A79I. Thus, we propose that our methodology can at least complement the consensus method. We also found that the criteria used to select mutants were important. In our first-round, we selected mutants based on amino acid position, considering only Tm but not affinity. If we had considered both Tm and affinity in the first-round, we likely would not have obtained A79I because A79F decreased affinity more than two-fold and would not have led to the second-round of selections at the A79 residue.

However, given that we identified only one mutant that simultaneously showed improved Tm and almost maintained affinity, we found it difficult to achieve both criteria at once even for single mutations not located in the paratope. We then conducted modeling and docking studies to investigate common characteristics of clones with improved Tm and clones with reduced affinity.

In our modeling study, we found that the structural changes induced by the point mutations were smaller than the difference between the wild type crystal structure and model structure. Nevertheless, comparison of the model structures allowed us to cluster them into two groups. Those in class-1 were closer in structure to the wild type VHH, while those in class-2 deviated from wild type structure. Further, while improvements in Tm were mostly observed in class-2, affinity maintenance was mostly observed in class-1 structures. From these analyses, we conclude that it is difficult to both increase the Tm and maintain affinity because of the conflicting effect of structural changes, which are desirable for increasing the Tm, but undesirable for maintaining affinity. Thus, classification of the overall structure in a modeling study was useful for interpreting our experimental data.

Here we discuss the first-round mutants (A24L, A79F, G97F and G26I) in more detail using model structures. The three sites, A24, A79, and G97, of the wild VHH, had a void above the side chain, and we predicted that filling the void with a hydrophobic amino acid would stabilize VHH in terms of dStability. Indeed, the mutant models showed that these new side chains filled the void. However, only A24 and A79 actually showed increased Tm, while G97 showed decreased Tm. Interestingly, G97 showed the lowest reduction in affinity in both SPR and ELISA. The model structures of A24L and A79F were assigned to class-2, while that of A79F was classified as class-1. Considering all of these findings, we speculate that the point mutation in A24L and A79F led to a conformational change to a class-2 structure, which filled the void to improve Tm, but reduced affinity for the antigen. In contrast, we speculate that although the void was also filled in G97F, its structure remained relatively unchanged; thus, antigen binding was maintained but the fine tuning required for stabilization did not occur. The G26I mutant also showed increased Tm, but, unlike A24L and A79F, its structure was classified under class-1, suggesting a different mechanism for the increased stability. In fact, the G26 residue is positioned such that if glycine is mutated into another amino acid, the side chain would point towards the outside of the protein. One possible cause of the increase in Tm may be that the mutation stabilized the dynamics of the loop structure. Detailed analysis using MD simulation is needed to confirm our hypothesis.

We then discuss the second-round mutants together with A79F in more detail using model structures. Interestingly, scatter plots of Tm versus affinity showed that similar amino acids, namely uncharged aromatic (F, W, Y), charged (H, R, E, K), aliphatic (I, L, V, M), uncharged polar (S, T, N, Q, C), and proline (P), tended to cluster together (Fig. 4b,c). The side chain of A79 extended into a space that was surrounded by the disulfide bond of C22–C96, the side chains of M34, W36, L51 and I70, and the backbone of T35 (Fig. 4d). Similar to A79F, mutants in which the residue at site A79 was substituted with two uncharged aromatic amino acids, W and Y, also showed an increase in Tm by more than 5 °C, although their affinity was reduced by more than two-fold. The dStability score predicts that substitutions to these uncharged aromatic amino acid residues would stabilize VHH by filling the voids, which was confirmed by the improvement in Tm. Mutation to these large side chains can be speculated to induce structural change from class-1 to class-2, as if a cardboard box is overstuffed and expanded. Such an environment would be enough to fill the void for enhancing Tm, but the structure change would decrease affinity for antigen.

The dStability score predicted that substitutions to mutants into charged amino acids, E, H, K and R, would destabilize VHH due to the low compatibility of charged amino acids with the mostly hydrophobic internal environment of the protein, and indeed our experimental values decreased Tm as anticipated. These four mutants also showed a greater reduction in affinity. This may be due to the large conformational change caused by the mutation, or because the equilibrium between folded and unfolded states tends to shift toward the unfolded state. Since the model structures of these mutants were classified as class-1, the conformational change was likely small. Therefore, it is possible that the equilibrium shifted from the folded state to the unfolded state due to instability within the protein in the folded state. This speculation is supported by the fact that there were insufficient amounts of the E and K mutant proteins to calculate K_D_ values. Meanwhile, histidine has two aspects one charged and one aromatic. According to Fig. 4a–c, histidine (H) is located near the charged residues, suggesting that the destabilizing effect of histidine’s electric charge was greater than the stabilizing effect of its aromatic group.

The dStability score also predicted that substitutions to aliphatic amino acid residues would stabilize VHH by filling the voids; however, A79I was the only mutant whose Tm increased as expected. Interestingly, the decrease in affinity was relatively small in mutants that received aliphatic amino acid substitution, especially A79I, A79V and A79L. I, L, and V are short aliphatic amino acids while M is a long aliphatic amino acid, and the model structures of A79I, A79V and A79L were grouped into class-1, while that of A79M was in class-2. These results indicate that substitutions with short aliphatic amino acids tend to maintain overall affinity because they do not greatly alter the structure. Further, the lack of structural change likely prevented the void from filling completely, which may explain why affinity did not improve except in the I mutant. In contrast, because methionine (M) is large in size, the mutation in A79M would have caused a conformational change into class-2, which, like the mutation into non-charged aromatic amino acids described above, resulted in a decrease in affinity. However, because methionine is less bulky than aromatic residues, it would not be able to fill the void, which explains the lack of increase in Tm.

Substitution with uncharged polar amino acids (S, T, N, Q, C) led to dStability scores and Tm values between those induced by substitution with hydrophobic and charged amino acids (Fig. 4a). The affinity of these mutants was also in-between those with hydrophobic and charged amino acid substitutions (Fig. 4b, c). Cysteine, which is the most hydrophobic of this group, may have contributed to the improved Tm by enhancing the hydrophobic environment in the protein core.

The dStability score predicted that substitutions to proline (P) would destabilize VHH, which was confirmed by our experiments. A proline side chain cannot fill the void around A79, which explains the lack of increase in Tm in A79P. Since the A79P model structure was classified as class-1, the reduction in affinity is likely unrelated to any conformational change, but instead may be the result of a shift in equilibrium from the folded to the unfolded structure. That we could only purify low amounts of the A79P mutant protein supports this speculation.

As noted above, the modeling study was effective for interpreting our experimental results. However, because there were significant differences between the crystal and model structures of the same wild type VHH, there are limitations to the above inferences. The docking study, on the other hand, was ineffective for interpreting the results of the present experiments. This is probably because the differences in the model structures of the point mutants were small, and the differences in the docking modes using these were even smaller and could not be detected. Using JOY to analyze local but distinct structural features in model structures, we found no distinct features between class-1 and class-2 structures, and no shared distinct features among mutants with increased Tm or unchanged affinity. It should be noted that this result does not reveal whether the point mutation was insufficient to change distinct features of the local structure or whether the lack of change is a consequence of poor modeling accuracy. More detailed structural information is needed to distinguish between the two possibilities. Especially, an X-ray crystal structure of the complex of the VHH mutant and the antigen is needed. In addition, as the present study was based on static structures, further analyses using MD simulations will be more informative.

In this paper, we used a simple and rapid experimental method. Finally, we discuss the problems caused by this. Our experiments to induce and examine the effects of a mutation comprised five steps: mutagenesis, plasmid DNA preparation, expression, purification, and Tm measurement, each of which consisted of minimal experiments. As the concentration and purity of plasmids were not uniform in our experiments, we discarded mutants in which the detected sequence differed from the target sequence without redoing the PCR. Further, while the target protein expression was confirmed by capillary electrophoresis in the two-round rational approach, the target protein expression was confirmed only by the concentration of the total purified protein rather than by capillary electrophoresis in the exhaustive alanine or valine scanning approach. Additionally, we measured Tm using DSF instead of DSC because the former allows for high throughput and use of low sample volumes. It is possible that by focusing on high throughput and low sample volume, we might have missed out on obtaining better mutants.

## Conclusion

We demonstrated that a comprehensive mutagenesis approach has low efficiency for identifying mutants with improved Tm. Instead, we propose a two-round rational approach using the dStability score. To our knowledge, this is the first study to show that a single mutation in VHH can increase the Tm by more than 5 °C while almost maintaining affinity. We speculate that the reason why it is difficult to simultaneously improve Tm and maintain affinity, even with single mutations not located in the paratope, is because of the conflicting effect of structural changes, which are desirable for increasing Tm, but undesirable for maintaining affinity. Given the inherent difficulty of improving Tm without reducing affinity, we recommend using a variety of approaches when trying to achieve such a feat. Our approach will be a useful complementary approach to other existing methods.

## Methods

### Calculation of dStability score

MOE molecular modeling software^28^ was used to display, analyze and manipulate protein structures, and to calculate dStability scores. We used the structure of the anti-hen egg lysozyme (HEL) VHH, D3-L11, complexed with its antigen (PDB Code 6JB8)^24,29^ in this study. After removing the antigen, water and ion molecules, leaving only VHH, we generated a side chain of N-terminal and C-terminal residues, and generated hydrogen atoms. D3-L11 is composed of 127 amino acids. Excluding both terminal residues, we mutated all 125 residues into 19 different amino acids. The 2,375 (125 × 19) total mutant structures were built using the Residue Scan function of MOE. Residue scan changes the amino acids and minimizes the energy of the mutated structure. The change in Gibbs free energy compared to the structure of wild type was calculated as dStability in MOE.

### VHH mutant preparation

The gene encoding the D3-L11 sequence with an N-terminal human immunoglobulin heavy chain signal peptide and C-terminal SAG tripeptide linker and 6 × His-tag was synthesized and cloned into the pcDNA3.4 expression vector by GeneArt (Life Technologies). Mutagenesis, protein expression and purification were performed in 96-well format. Site-directed mutagenesis was conducted by inverse PCR using PrimeSTAR Max DNA Polymerase (Takara Bio, Cat. No. R045A) under the following PCR conditions: pre-denaturation at 98 °C for 1 min; 30 cycles of denaturation at 98 °C for 10 s, annealing at 55 °C for 15 s and amplification at 72 °C for 55 s. Primer pairs used in the inverse PCR reaction to introduce each mutation are designed according to the manufacturer’s instructions.

The PCR products were directly transformed into *E.coli* JM109 competent cells (Takara Bio, Cat. No. 9052). The transformed cells were cultured at 37 °C overnight in LB/Ampicillin media, and the mutated plasmids were prepared using a Wizard MagneSil Tfx kit (Promega, Cat. No. A2380). Mutant sequences were confirmed by standard Sanger sequencing (3730xl Genetic Analyzer, Applied Biosystems). Wild type and mutant VHH proteins were expressed by transient transfection using the ExpiCHO Expression System (Thermo Fisher Scientific, Cat. No. A29133) according to manufacturer’s High Titer protocol. The cells were cultured for 10 days at 32ºC with 5% CO_2_. Supernatants were collected by centrifugation and loaded into His Mag Sepharose excel (GE Healthcare, Cat No. 17371221) equilibrated with binding buffer (20 mM Tris/HCl pH 7.5, 500 mM NaCl). His Mag Sepharose excel was washed three times with 300 µL of wash buffer (20 mM Tris/HCl pH 7.5, 500 mM NaCl, 20 mM imidazole). VHHs were eluted with 140 µL of elution buffer (20 mM Tris/HCl pH 7.5, 500 mM NaCl, 500 mM imidazole) and the buffer was changed to PBS using PD MultiTrap G-25 (GE Healthcare, Cat. No. 28918006) according to manufacturer’s protocol. Protein concentration was determined by UV (280 nm) absorption using a NanoDrop 8000 spectrophotometer (Thermo Fisher Scientific). The molecular weight and purity of each purified VHH was analyzed by capillary electrophoresis using LabChip GXII Touch HT (PerkinElmer) according to the manufacturer’s protocol for protein analysis under reducing conditions.

### Differential scanning fluorometry

Purified VHH samples whose concentration was higher than 0.35 mg/mL were diluted to 0.35 mg/mL with PBS. Purified VHH samples whose concentration was less than 0.35 mg/mL were used without dilution. 9 µL volume of diluted or stock solution was mixed with 1 µL of 100-fold diluted SYPRO^®^ Orange Protein Gel Stain (Life Technologies, Cat No. S6650). DSF was performed in 384-well PCR plates using CFX384 Touch Real-Time PCR systems (Bio-Rad Laboratories). The fluorescence intensity (Ex/Em = 470/570 nm) of SYPRO^®^ Orange was monitored while increasing the temperature from 25 °C to 95 °C at 0.5 °C increments every 20 s. The melting temperature (Tm) was analyzed using CFX Maestro software (Bio-Rad Laboratories). Measurements were performed in duplicate and average values are plotted in Fig. 1 and Fig. 3a.

### Surface plasmon resonance (SPR)

The affinity of D3-L11 to lysozyme was determined by SPR using a Biacore T200 (GE Healthcare). Lysozyme was immobilized on the surface of the Sensor Chip CM5 using standard amine coupling to a level of 200 response units (RU). Analysis was performed by injecting the VHH samples onto the CM5 surface for 300 s to measure the RU in the association reaction and then injecting a continuous flow of PBST (PBS supplemented with 0.005% Tween 20) running buffer for 600 s to measure the RU in the dissociation reaction. Regeneration was performed by injecting 1.0 M arginine-HCl (pH 4.4) buffer to remove the bound antibodies on the CM5 surface and then equilibrating the sensor chip with PBST before analyzing the next antibody sample. Measurements were performed in duplicate and a sensorgram of RU against time was plotted to show changes in the interaction. The kinetic parameters kon, koff, and K_D_ of the VHHs against the lysozyme were calculated using a 1:1 binding model in BiaEvaluation Software.

### Enzyme-linked immunosorbent assay (ELISA)

A 384-well MaxiSorp plate (Thermo Fisher Scientific, Cat No. 464718) was coated with 20 µL/well of lysozyme (1 µg/mL, Sigma-Aldrich, Cat No. L6876) and incubated overnight at 4 °C. After removal of lysozyme solution, the plate was blocked with 50 µL/well of 20% BlockingOne (Nacalai Tesque, Cat No. 03953-95) in TBST buffer (25 mM Tris/HCl pH 7.4, 137 mM NaCl, 2.68 mM KCl, 0.05% Tween 20, v/v) for 1.5 h at room temperature. After washing the plate with 100 μL of TBST buffer, 20 μL of each serial concentration of VHH diluted with 5% BlockingOne in TBST buffer was added to each well of the plate. After incubating for 1 h at room temperature and washing three times with TBST, 20 μL of 2000-fold diluted Penta-His HRP conjugate (Qiagen, Cat No. 34460) was added to each well. The plate was then incubated for 1 h at room temperature.

After washing three times with TBST, 20 μL of TMB + One-Step Substrate System (Dako, Cat No. S1601) was added to each well, and the plate was incubated for 5 min at room temperature. Enzyme reactivity was terminated by adding 20 μL of 1 M H_2_SO_4_. Absorbance was measured using Infinite M200pro (Tecan) at 450 nm with reference absorbance at 570 nm. ELISA data were analyzed using GraphPad Prism 8.

### Modeling and docking simulations

The 3D models of first and second-round mutants and wild type were constructed using the Antibody Modeler function in MOE, based on the D3-L11 structure in the complex crystal structure of wild type D3-L11 and lysozyme (PDB ID: 6JB8). This structure was automatically selected as a template by the software when we selected the apo D3-L11 structure (PDB ID: 6JB9) as a query. After energy minimization without fixing the main chain, we calculated the RMSD of the full-length main chain between wild type and each mutant.

After aligning the sequences and structures of these 3D models using MOE, we extracted structural information such as the secondary structure, accessible surface area, hydrogen bonds to the main chain carbonyl/amide, disulfide bonds and phi angle using JOY^30^ and exported the annotations.

Docking simulations of the interaction between the model structures of VHH and the crystal structure of the antigen lysozyme were performed using FRODOCK 3.12^31,32^ without restrictions on the binding interface between VHH and lysozyme according to the default setting in the distributed script. The lysozyme and wild type D3-L11 structures were obtained from the complex structure of D3-L11 and lysozyme (PDB ID: 6JB8). For the structure of the mutant, we used the model structure described previously in this section. For each calculation, the docking pose with the highest score was selected. In each docking pose, we tabulated the number of interactions between each of the 11 residues of the paratope (E32, Y52, H54, T55, K101, Y102, P104, R106, F107, S113 and D115) and the antigen lysozyme for each type of interaction (hydrophobic interaction, ionic interaction and hydrogen bonding).

## Supplementary Information

Supplementary Information 1.

Supplementary Information 2.

## Data Availability

The datasets generated and/or analyzed during the current study are available from the corresponding author on reasonable request.
