# Supplementary material for: Increasing the melting temperature of VHH with the in silico free energy score
Source: Sci Rep. 2023 Mar 25;13:4922. doi: 10.1038/s41598-023-32022-8 (PMC10039853; doi:10.1038/s41598-023-32022-8)
Supplement: Supplementary file 1 — Supplementary Information 1. [file 41598_2023_32022_MOESM1_ESM.pdf]

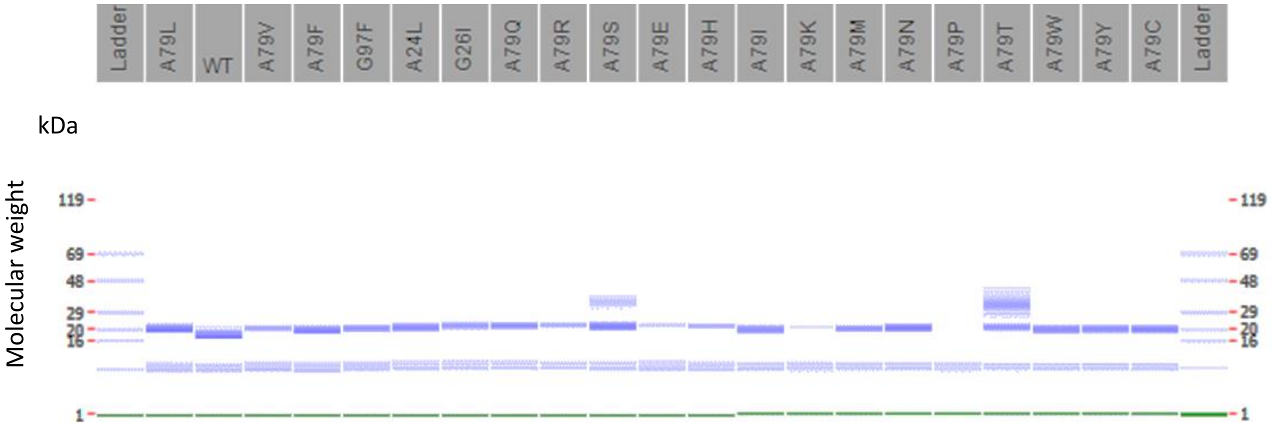

Supplementary Figure S1

Capillary electrophoresis image of wild type and first-/second-round mutants. This image was obtained by integrating the electropherograms of each VHHs. Ladder lane indicates molecular weight standard.

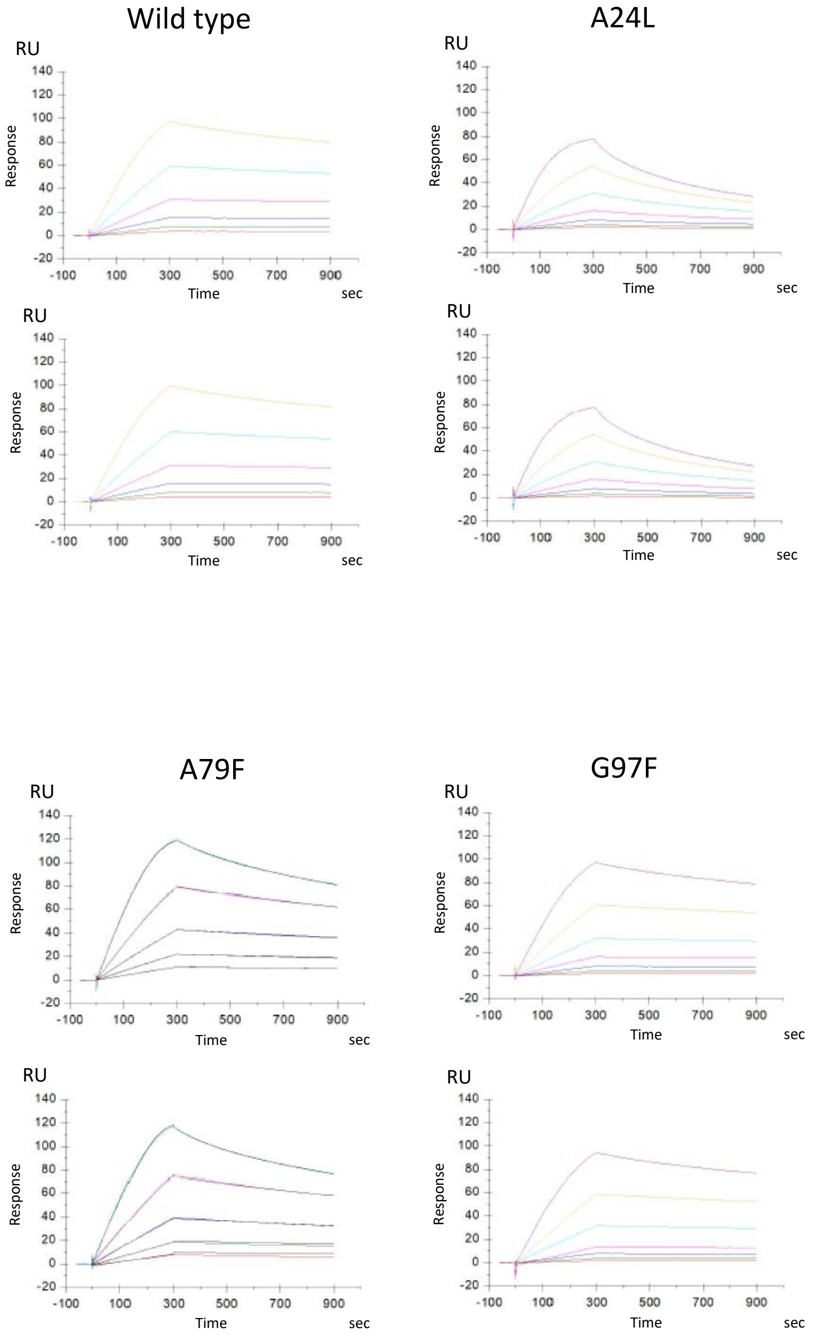

Supplementary Figure S2

SPR sensorgrams of wild type and first-round mutants. Each measurement was performed in duplicate.

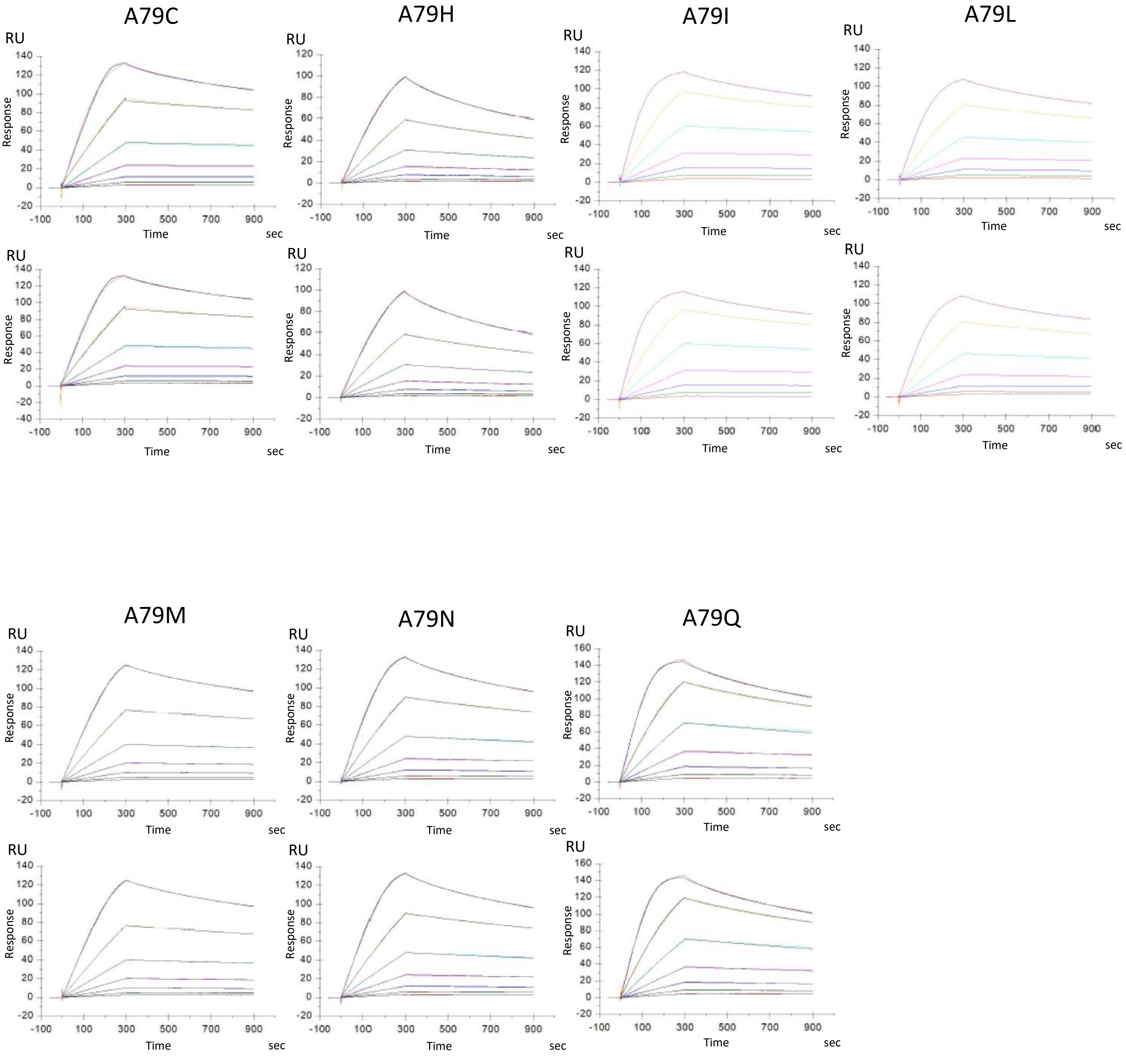

Supplementary Figure S3
SPR sensorgrams of second-round mutants. Each measurement was performed in duplicate.

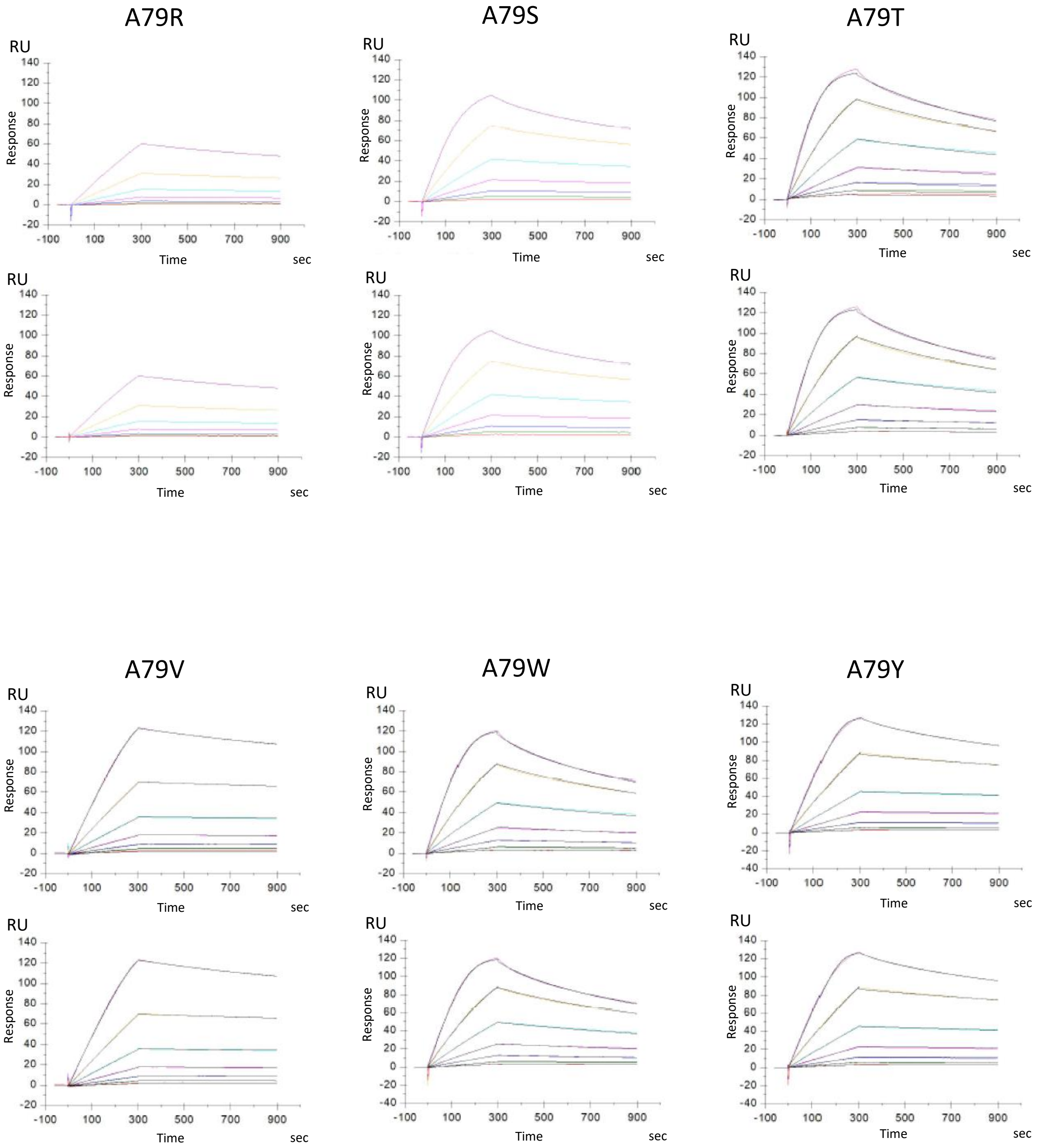

Supplementary Figure S4

SPR sensorgrams of second-round mutants. Each measurement was performed in duplicate.

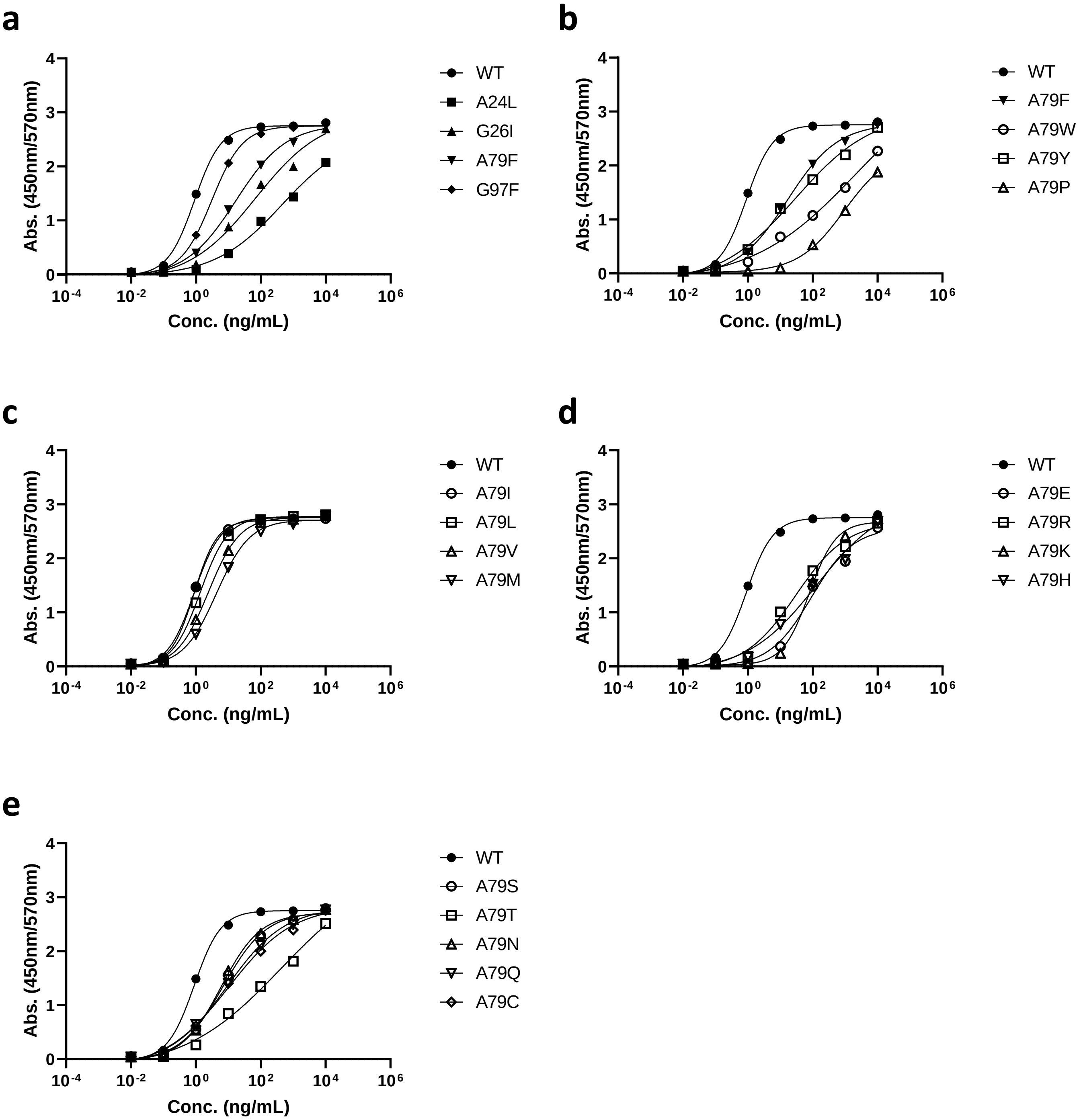

Supplementary Figure S5

ELISA binding curves of wild type and each mutant. The wild type binding curve is included in each graph for comparison. (a) Binding curves of the 4 mutants selected in the first round. (b) Binding curves of mutants generated by substituting the residue at A79 with aromatic residues and proline. (c) Binding curves of mutants generated by substituting the residue at A79 with aliphatic residues. The curve of A79I overlaps with that of the wild type. (d) Binding curves of mutants generated by substituting the residue at A79 with charged residues. (e) Binding curves of mutants generated by substituting the residue at A79 with uncharged polar residues.

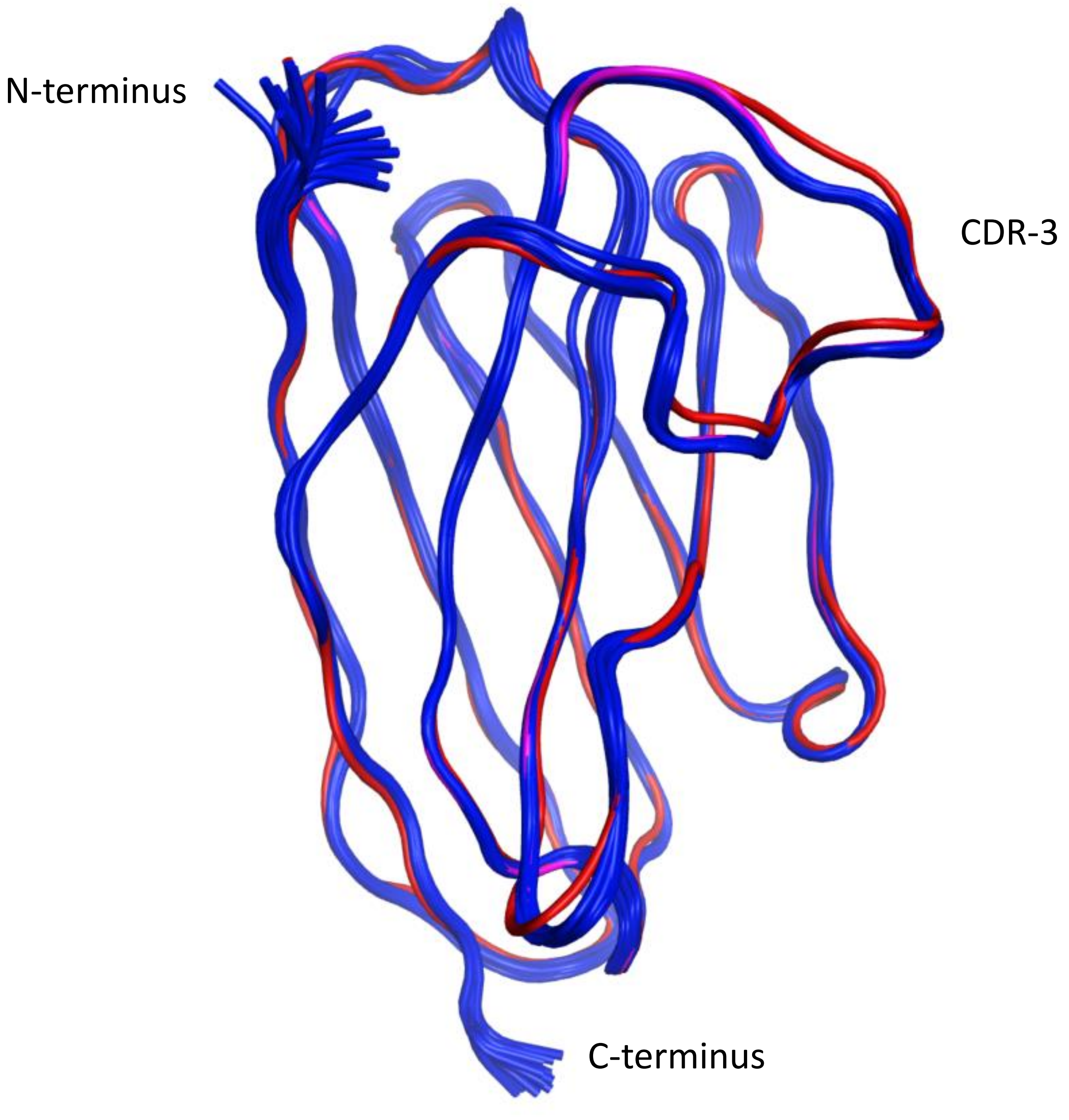

Supplementary Figure S6
Ribbon diagram of the first and second-round mutants and the wild type model structure and wild type crystal structure (PDB ID: 6JB9). Wild type crystal structure and model structure are shown in red and magenta, respectively. The mutant model structures are shown in blue.

	WT 6JB9	WT model	G26I	G97F	A24L	A79F	A79Y	A79W	A79M	A79Q	A79S	A79I	A79C	A79L	A79V	A79H	A79E	A79K	A79R	A79T	A79N	A79P
WT (6JB9)	0	0.92	0.9	0.91	0.88	0.9	0.85	0.91	0.83	0.8	0.86	0.94	0.93	1.03	0.86	0.92	0.95	0.93	0.96	1.03	1	0.93
WT (model)	0.92	0	0.28	0.4	0.62	0.72	0.62	0.67	0.6	0.61	0.56	0.32	0.31	0.42	0.33	0.34	0.3	0.32	0.37	0.28	0.29	0.41
G26I	0.9	0.28	0	0.44	0.61	0.75	0.62	0.68	0.61	0.61	0.57	0.41	0.42	0.56	0.34	0.36	0.34	0.38	0.45	0.36	0.36	0.38
G97F	0.91	0.4	0.44	0	0.69	0.69	0.69	0.73	0.61	0.67	0.65	0.45	0.46	0.46	0.45	0.32	0.47	0.34	0.44	0.49	0.45	0.52
A24L	0.88	0.62	0.61	0.69	0	0.57	0.39	0.58	0.45	0.4	0.34	0.65	0.64	0.75	0.42	0.62	0.67	0.67	0.69	0.69	0.7	0.52
A79F	0.9	0.72	0.75	0.69	0.57	0	0.44	0.53	0.37	0.42	0.43	0.65	0.67	0.64	0.6	0.65	0.72	0.62	0.65	0.76	0.72	0.7
A79Y	0.85	0.62	0.62	0.69	0.39	0.44	0	0.45	0.36	0.35	0.34	0.59	0.59	0.72	0.46	0.63	0.62	0.62	0.62	0.69	0.67	0.53
A79W	0.91	0.67	0.68	0.73	0.58	0.53	0.45	0	0.49	0.5	0.44	0.67	0.68	0.76	0.56	0.73	0.65	0.67	0.7	0.7	0.72	0.63
A79M	0.83	0.6	0.61	0.61	0.45	0.37	0.36	0.49	0	0.26	0.31	0.6	0.61	0.63	0.44	0.56	0.64	0.53	0.54	0.66	0.67	0.58
A79Q	0.8	0.61	0.61	0.67	0.4	0.42	0.35	0.5	0.26	0	0.28	0.61	0.62	0.68	0.47	0.63	0.65	0.61	0.61	0.68	0.68	0.6
A79S	0.86	0.56	0.57	0.65	0.34	0.43	0.34	0.44	0.31	0.28	0	0.57	0.57	0.66	0.42	0.61	0.57	0.58	0.59	0.61	0.61	0.52
A79I	0.94	0.32	0.41	0.45	0.65	0.65	0.59	0.67	0.6	0.61	0.57	0	0.17	0.42	0.42	0.39	0.3	0.37	0.43	0.37	0.29	0.42
A79C	0.93	0.31	0.42	0.46	0.64	0.67	0.59	0.68	0.61	0.62	0.57	0.17	0	0.42	0.43	0.41	0.33	0.38	0.41	0.37	0.31	0.45
A79L	1.03	0.42	0.56	0.46	0.75	0.64	0.72	0.76	0.63	0.68	0.66	0.42	0.42	0	0.53	0.42	0.49	0.33	0.35	0.41	0.39	0.58
A79V	0.86	0.33	0.34	0.45	0.42	0.6	0.46	0.56	0.44	0.47	0.42	0.42	0.43	0.53	0	0.37	0.41	0.41	0.43	0.43	0.45	0.33
A79H	0.92	0.34	0.36	0.32	0.62	0.65	0.63	0.73	0.56	0.63	0.61	0.39	0.41	0.42	0.37	0	0.41	0.25	0.35	0.43	0.37	0.43
A79E	0.95	0.3	0.34	0.47	0.67	0.72	0.62	0.65	0.64	0.65	0.57	0.3	0.33	0.49	0.41	0.41	0	0.37	0.45	0.34	0.27	0.43
A79K	0.93	0.32	0.38	0.34	0.67	0.62	0.62	0.67	0.53	0.61	0.58	0.37	0.38	0.33	0.41	0.25	0.37	0	0.27	0.38	0.33	0.49
A79R	0.96	0.37	0.45	0.44	0.69	0.65	0.62	0.7	0.54	0.61	0.59	0.43	0.41	0.35	0.43	0.35	0.45	0.27	0	0.4	0.42	0.52
A79T	1.03	0.28	0.36	0.49	0.69	0.76	0.69	0.7	0.66	0.68	0.61	0.37	0.37	0.41	0.43	0.43	0.34	0.38	0.4	0	0.28	0.45
A79N	1	0.29	0.36	0.45	0.7	0.72	0.67	0.72	0.67	0.68	0.61	0.29	0.31	0.39	0.45	0.37	0.27	0.33	0.42	0.28	0	0.45
A79P	0.93	0.41	0.38	0.52	0.52	0.7	0.53	0.63	0.58	0.6	0.52	0.42	0.45	0.58	0.33	0.43	0.43	0.49	0.52	0.45	0.45	0

Supplementary Figure S7

RMSD matrix of model structures. Each RMSD value (Unit: Å) indicates the difference in the position of the main chain atom. WT (6JB9) shows the crystal structure of wild type D3-L11 (PDB ID: 6JB9). WT (model) shows the model structure of wild type D3-L11 using the VHH structure in the complex crystal structure (PDB ID: 6JB8) as a template.

	10 20 30 40 50
WT (6JB9)	dVqLvEsgggsVqaggsLrLsCaAsgstdsIeYMTWFRqApgkareGVAA
WT (model)	DvqLvEsgggsVqaggsLrLSCaAsgstdsIEYMTWFRqApgkareGVAA
G26I	DvqLvEsgggsvqaggsLrLSCaAsistdsIEYMTWFRqApgkareGVAA
G97F	DvqLvesgggsvqaggsLrLSCaAsgstdsIEYMTWFRqApgkareGVAA
A24L	dvqLvEsgggsvqagGsLrLSCaLsgstdsIEYMTWFRqApgkareGVAA
A79F	dVqLvEsgggsVqaggsLrLSCaAsgstdsIEYMTWFRqApgkareGVAA
A79Y	dVqLvEsgggsvqaggsLrLSCaAsgstdsIEYMTWFRqApgkareGVAA
A79W	dVqLvEsgggsvqaggsLrLSCaAsgstdsIEYMTWFRqApgkareGVAA
A79M	dVqLvEsgggsvqaggsLrLSCaAsgstdsIEYMTWFRqApgkareGVAA
A79Q	dVqLvesgggsvqaggsLrLSCaAsgstdsIEYMTWFRqApgkareGVAA
A79S	dVqLvEsgggsvqaggsLrLSCaAsgstdsIEYMTWFRqApgkareGVAA
A79I	DvqLvEsgggsvqaggsLrLSCaAsgstdsIEYMTWFRqApgkareGVAA
A79C	DvqLvEsgggsvqaggsLrLSCaAsgstdsIEYMTWFRqApgkareGVAA
A79L	DvqLvEsgggsVqaggsLrLSCaAsgstdsIEYMTWFRqApgkareGVAA
A79V	dvqLvEsgggsvqaggsLrLSCaAsgstdsIEYMTWFRqApgkareGVAA
A79H	DvqLvEsgggsVqaggsLrLSCaAsgstdsIEYMTWFRqApgkareGVAA
A79E	DvqLvEsgggsVqaggsLrLSCaAsgstdsIEYMTWFRqApgkareGVAA
A79K	DvqLvEsgggsVqaggsLrLSCaAsgstdsIEYMTWFRqApgkareGVAA
A79R	DvqLvEsgggsVqaggsLrLSCaAsgstdsIEYMTWFRqApgkareGVAA
A79T	DvqLvEsgggsVqaggsLrLSCaAsgstdsIEYMTWFRqApgkareGVAA
A79N	DvqLvEsgggsVqaggsLrLSCaAsgstdsIEYMTWFRqApgkareGVAA
A79P	DvqLvEsgggsvqaggsLrLSCaAsgstdsIeYMTWFRqApgkareGVAA
	bbb bbb bbbbbb bbbbbbb bbbbb

● paratope

solvent inaccessible (ASA < 0.1)	UPPER CASE	X
solvent accesible	lower case	x
alpha helix	red	x
beta strand	blue	x
3_{10} helix	maroon	x
hydrogen bond to main-chain amide	**bold**	**x**
hydrogen bond to main-chain carbonyl	underline	x
disulfide bond	cedilla	ç
positive phi torsion angle	*italic*	*x*

Supplementary Figure S8a
Sequence alignment of wild type and each mutant using JOY format.

	60 70 80 90 100
WT (6JB9)	LyThtgntyytdsVkgrFtIsqdkaknmAyLrMdsVkseDtAiYTCGATr
WT (model)	LyThtgntyytdsVkgrFtIsqdkakNmAyLrMdsVkseDtAiYTCGATr
G26I	LyThtgntyytdsVkgrFtIsqdkakNmAyLrMdsVkseDtAiYTCGATr
G97F	LyThtgntyytdsVkgrFtIsqdkakNmAyLrMdsVkseDtAiYTCFATr
A24L	LyThtgntyytdsVkgrFtIsqdkakNmAyLrMdsVkseDtAiYTCGATr
A79F	LyThtgntyytdsVkgrFtIsqdkakNmFyLrMdsVkseDtAiYTCGATr
A79Y	LyThtgntyytdsVkgrFtIsqdkakNmYyLrMdsVkseDtAiYTCGATr
A79W	LyThtgntyytdsVkgrFtIsqdkakNmWyLrMdsVkseDtAiYTCGATr
A79M	LyThtgntyytdsVkgrFtIsqdkakNmMyLrMdsVkseDtAiYTCGATr
A79Q	LyThtgntyytdsVkgrFtIsqdkakNmQyLrMdsVkseDtAiYTCGATr
A79S	LyThtgntyytdsVkgrFtIsqdkakNmSyLrMdsVkseDtAiYTCGATr
A79I	LyThtgntyytdsVkgrFtIsqdkakNmIyLrMdsVkseDtAiYTCGATr
A79C	LyThtgntyytdsVkgrFtIsqdkakNmCyLrMdsVkseDtAiYTCGATr
A79L	LyThtgntyytdsVkgrFtIsqdkakNmLyLrMdsVkseDtAiYTCGATr
A79V	LyThtgntyytdsVkgrFtIsqdkakNmVyLrMdsVkseDtAiYTCGATr
A79H	LyThtgntyytdsVkgrFtIsqdkakNmHyLrMdsVkseDtAiYTCGATr
A79E	LyThtgntyytdsVkgrFtIsqdkakNmEyLrMdsVkseDtAiYTCGATr
A79K	LyThtgntyytdsVkgrFtIsqdkakNmKyLrMdsVkseDtAiYTCGATr
A79R	LyThtgntyytdsVkgrFtIsqdkakNmRyLrMdsVkseDtAiYTCGATr
A79T	LyThtgntyytdsVkgrFtIsqdkakNmTyLrMdsVkseDtAiYTCGATr
A79N	LyThtgntyytdsVkgrFtIsqdkakNmNyLrMdsVkseDtAiYTCGATr
A79P	LyThtgntyytdsVkgrFtIsqdkakNmPyLrMdsVkseDtAiYTCGATr
	bb bbb bbbbbb bbbbbb 333 bbbbbb
	● ●●

● paratope

solvent inaccessible (ASA < 0.1)	UPPER CASE	X
solvent accesible	lower case	x
alpha helix	red	x
beta strand	blue	x
3_{10} helix	maroon	x
hydrogen bond to main-chain amide	**bold**	**x**
hydrogen bond to main-chain carbonyl	underline	x
disulfide bond	cedilla	ç
positive phi torsion angle	*italic*	*x*

Supplementary Figure S8b
Sequence alignment of wild type and each mutant using JOY format.

```
                  110       120
WT (6JB9)   kyvpvrfAldqsSYdywgqgTqVtVss
WT (model)  kyVpvrfAldqssYdywgqgtqVtVss
G26I        kyVpvrfAldqssYdywgqgtqVtVss
G97F        kyVpvrfAldqssYdywgqgtqVtVss
A24L        kyVpvrfAldqssYdywgqgtqVtVss
A79F        kyVpvrfAldqssYdywgqgtqVtVss
A79Y        kyVpvrfAldqssYdywgqgtqVtVss
A79W        kyVpvrfAldqssYdywgqgtqVtVss
A79M        kyVpvrfAldqssYdywgqgtqVtVss
A79Q        kyVpvrfAldqssYdywgqgtqVtVss
A79S        kyVpvrfAldqssYdywgqgtqVtVss
A79I        kyVpvrfAldqssYdywgqgtqVtVss
A79C        kyVpvrfAldqssYdywgqgtqVtVss
A79L        kyVpvrfAldqssYdywgqgtqVtVss
A79V        kyVpvrfAldqssYdywgqgtqVtVss
A79H        kyVpvrfAldqssYdywgqgtqVtVss
A79E        kyVpvrfAldqssYdywgqgtqVtVss
A79K        kyVpvrfAldqssYdywgqgtqVtVss
A79R        kyVpvrfAldqssYdywgqgtqVtVss
A79T        kyVpvrfAldqssYdywgqgtqVtVss
A79N        kyVpvrfAldqssYdywgqgtqVtVss
A79P        kyVpvrfAldqssYdywgqgtqVtVss
                333      bb    bbb
```

● paratope

solvent inaccessible (ASA < 0.1)	UPPER CASE X
solvent accesible	lower case x
alpha helix	red x
beta strand	blue x
3_{10} helix	maroon x
hydrogen bond to main-chain amide	**bold** **x**
hydrogen bond to main-chain carbonyl	underline x
disulfide bond	cedilla ç
positive phi torsion angle	*italic* *x*

Supplementary Figure S8c
Sequence alignment of wild type and each mutant using JOY format.

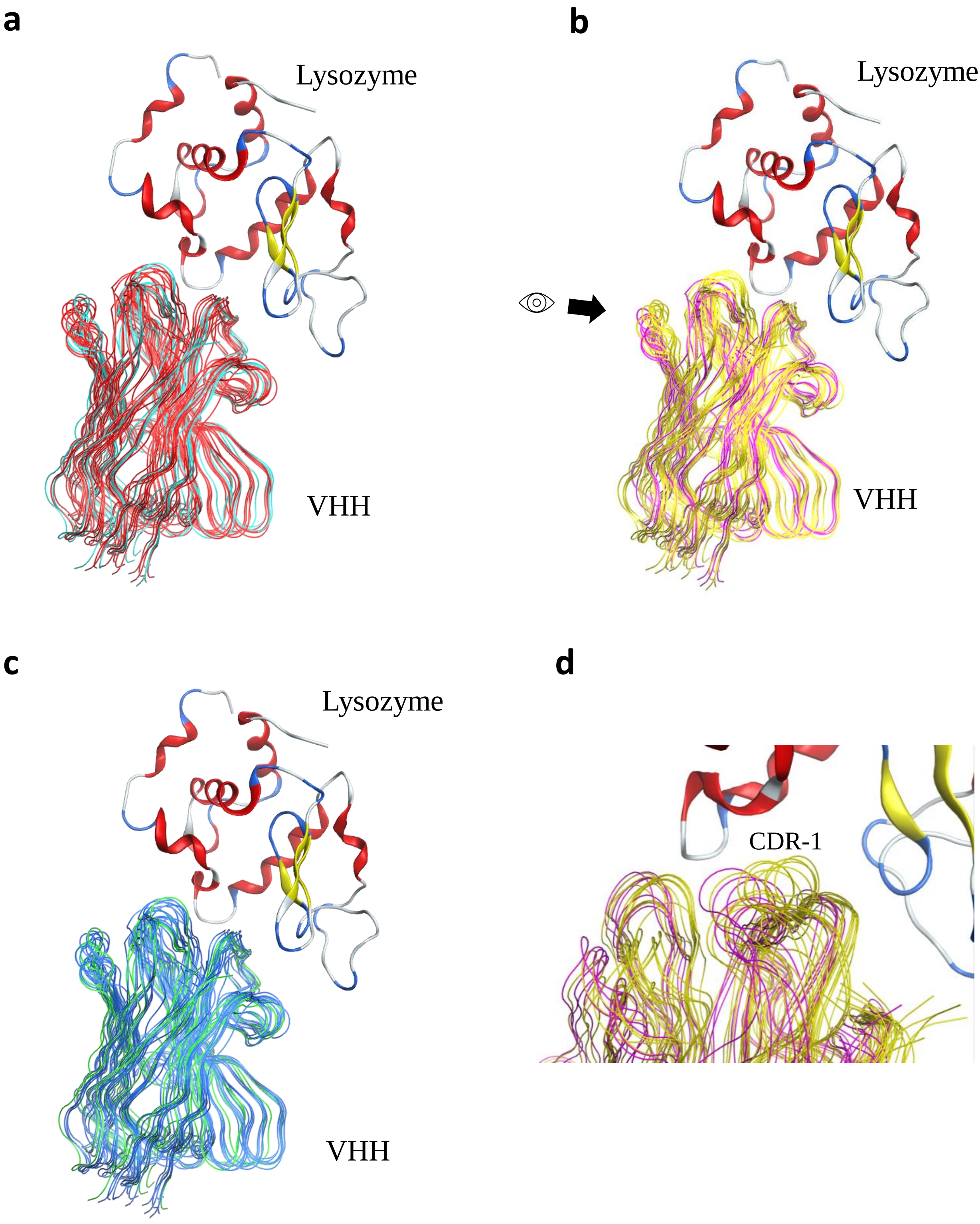

Supplementary Figure S9

Superposed docking poses with the best docking score of wild type with each mutant. The lysozyme is depicted in the ribbon diagram. The color scheme differs in each figure as follows:

(a) Class-1 and class-2 docking poses are shown by the red line and cyan line, respectively.

(b) Class-1 and class-2 docking poses are shown by the magenta line and yellow line, respectively. The viewpoints in Supplementary Figure S7d are indicated by arrows.

(c) Mutants with Tm increases higher than 5° C and others are shown by the green line and blue line, respectively.

(d) Close-up view of docking poses classified as class-1 and class-2. Color scheme is the same as that in Supplementary Figure S7b.

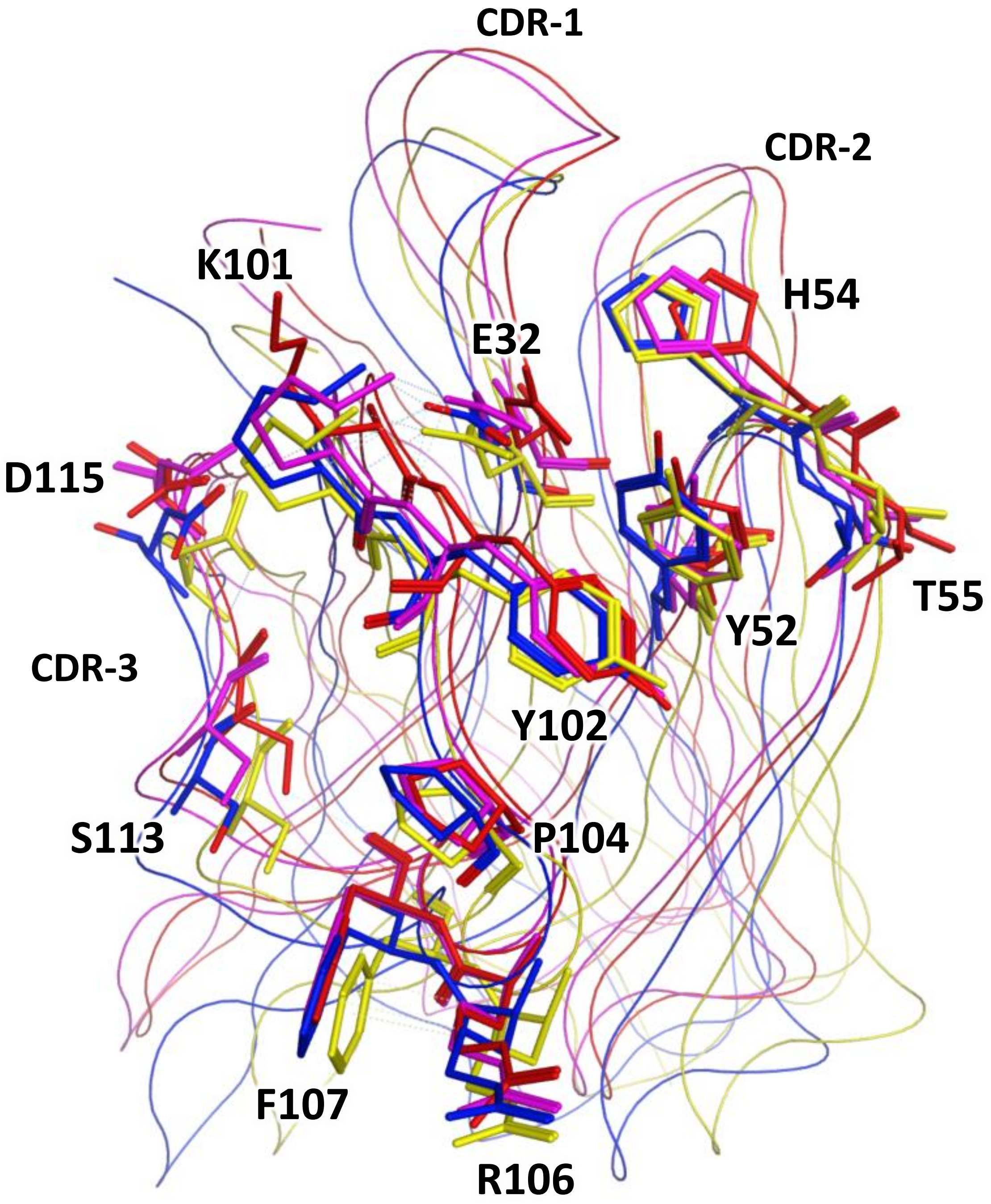

Supplementary Figure S10

Superposed docking structures. Wild type crystal structure, wild type model structure, A79I and A79W mutants are colored red, magenta, yellow and blue, respectively. Main chains are depicted as lines, and paratope residues are depicted as sticks.
